# Antibiotic prescribing behavior among general practitioners – a questionnaire-based study in Germany

**DOI:** 10.1186/s12879-018-3120-y

**Published:** 2018-05-04

**Authors:** Florian Salm, Sandra Schneider, Katja Schmücker, Inga Petruschke, Tobias S. Kramer, Regina Hanke, Christin Schröder, Christoph Heintze, Ulrich Schwantes, Petra Gastmeier, Jochen Gensichen, on behalf of the RAI-Study Group, Muna Abu Sin, Esther-Maria Antao, Michael Behnke, Evgeniya Boklage, Tim Eckmanns, Christina Forstner, Petra Gastmeier, Jochen Gensichen, Alexander Gropmann, Stefan Hagel, Regina Hanke, Wolfgang Hanke, Anja Klingeberg, Lukas Klimmek, Ulrich Kraft, Markus Lehmkuhl, Norman Ludwig, Antina Lu¨bke-Becker, Oliwia Makarewicz, Anne Moeser, Inga Petruschke, Mathias W. Pletz, Florian Salm, Katja Schmücker, Sandra Schneider, Christin Schröder, Frank Schwab, Joachim Trebbe, Szilvia Vincze, Horst Christian Vollmar, Jan Walter, Sebastian Weis, Wibke Wetzker, Lothar H. Wieler

**Affiliations:** 1 Institute for Infection Prevention and Hospital Epidemiology, Medical Center – University of Freiburg, Breisacher Str. 115 B, D-79106 Freiburg, Germany; 2 Institute of Hygiene and Environmental Medicine, Charité University Medical Center Berlin, German National Reference Center for the Surveillance of Nosocomial Infections, Hindenburgdamm 27, D-12203 Berlin, Germany; 3 Institute of General Practice and Family Medicine, Jena University Hospital, Friedrich-Schiller-University, School of Medicine, Bachstrasse 18, D-07743 Jena, Germany; 4 Lindgrün GmbH, Cuxhavener Strasse 12, D-10555 Berlin, Germany; 5 Institute Institute for General Practice and Family Medicine, Charité University Medical Center Berlin, Charitéplatz 1, D-10117 Berlin, Germany; 6 Medizinische Hochschule Brandenburg Theodor Fontane, Fehrbelliner Str. 38, D-16816 Neuruppin, Germany; 7 Institute for General Practice, Ludwig-Maxilmilians-University/University Hospital, Pettenkofer str.8/10, D-80336 Munich, Germany

**Keywords:** Antibiotic therapy, Primary care, Antimicrobial resistance, Antibiotic policy

## Abstract

**Background:**

This study investigates the barriers and facilitators of the use of antibiotics in acute respiratory tract infections by general practitioners (GPs) in Germany.

**Methods:**

A multidisciplinary team designed and pre-tested a written questionnaire addressing the topics *awareness of antimicrobial resistance* (7 items), *use of antibiotics* (9 items), *guidelines/sources of information* (9 items) and *sociodemographic factors* (7 items), using a five-point-Likert-scale (“never” to “very often”). The questionnaire was mailed by postally to 987 GPs with registered practices in eastern Germany in May 2015.

**Results:**

34% (340/987) of the GPs responded to this survey. Most of the participants assumed a multifactorial origin for the rise of multidrug resistant organisms. In addition, 70.2% (239/340) believed that their own prescribing behavior influenced the drug-resistance situation in their area. GPs with longer work experience (> 25 years) assumed less individual influence on drug resistance than their colleagues with less than 7 years experience as practicing physicians (Odds Ratio [OR] 0.32, 95% Confidence Interval [CI] 0.17–0.62; *P* < 0.001). 99.1% (337/340) of participants were familiar with the “delayed prescription” strategy to reduce antibiotic prescriptions. However, only 29.4% (74/340) answered that they apply it “often” or “very often”. GPs working in rural areas were less likely than those working in urban areas to apply delayed prescription.

**Conclusion:**

The knowledge on factors causing antimicrobial resistance in bacteria is good among GPs in eastern Germany. However measures to improve rational prescription are not widely implemented yet. Further efforts have to be made in order to improve rational prescription of antibiotic among GPs. Nevertheless, there is a strong awareness of antimicrobial resistance among the participating GPs.

**Electronic supplementary material:**

The online version of this article (10.1186/s12879-018-3120-y) contains supplementary material, which is available to authorized users.

## Background

Antimicrobial resistance (AMR) jeopardizes the achievements of modern medicine in Europe and worldwide [1–3]. The consumption of antibiotics is an important driver of AMR [4, 5]. Over the past decade the global antibiotic use increased significantly [6]. In the human sector the primary use of antibiotics in outpatient care is found among general practitioners (GPs) [7, 8]. There is a considerable difference in outpatient antibiotic use worldwide, between European countries and within countries [9–11]. Considering the use of antibiotics in primary care in Europe, Germany is one of the countries with a lower level of consumption of antibiotics [12, 13]. Nevertheless, it is striking that the proportion of reserve antibiotics in Germany is high [11, 14]. In Germany, the total consumption of antibiotics in human medicine is about 800 tons per year. Approximately 600 tons of this are used in outpatient care [15]. More than half of the antibiotics used in outpatient care are prescribed by GPs in Germany [16, 17]. In GP practices, the majority of antibiotics is prescribed for acute respiratory infections, most of which are caused by a virus [16, 18]. In most cases they do not require antimicrobial therapy [19–22]. In Germany, antimicrobial resistance is mainly a problem in hospital care and especially in intensive care units [23]. German health care system is divided in primary and secondary care, most people are covered by statutory health insurance [24].

The present survey was carried out in the preparation of a broader intervention study, called “*Rational antibiotic Use via information and communication*” (RAI-project, www.rai-projekt.de). The RAI-project promotes rational antibiotic use in veterinary medicine, in particular in pig farming, as well as in human medicine, surgery and intensive care units, travel medicine, and primary care, with a focus on eastern Germany [25].

The following barriers to rational antibiotic usage have been identified from the scientific literature. But explanations of the barriers to rational antibiotic use vary widely in primary care. Some authors describe that uncertainty about pathogenesis, heavy workflow and patient’s desire for an antibiotic therapy can lead to increased prescription of antibiotics [26–28] as well as knowledge and health literacy among the general population [29]. This questionnaire survey was carried out to examine whether the barriers identified in the literature can also be found in the intervention area.

## Methods

### Survey development

A multidisciplinary team of the RAI study group developed a questionnaire comprised of 32 questions grouped around the four issues: *awareness of antimicrobial resistance* (7 items), *use of antibiotics* (9 items), *guidelines/sources of information* (9 items) and *socio-demographic factors* (7 items). The majority of the answers were in tick-box format (see Additional file 1).

To identify factors influencing prescribing behavior of antibiotics we conducted a literature review. Based on these results, the questionnaire was developed. The inquiry of the sociodemographic data (Q1-Q4), as well as the questions Q8, Q9, Q12 and Q13 are founded on a previous study conducted among GPs in Germany in 2007 [30].

The questionnaire was pretested among scientists at the Friedrich-Schiller-University (Jena) and the Charité (Berlin). In a second step, a pilot test was conducted among 12 GPs (03/2015). After completing the questionnaire, participants were asked to explain the content of each question in their own words to increase internal validity. GPs who took part in the pilot test were not included in the survey.

### Recruitment and data collection

The revised questionnaire was then mailed postally to roughly one third (987) of the GPs from the German federal states of Thuringia, Brandenburg and Berlin (2015/05).

In Berlin and Thuringia, pre-existing lists of all registered doctors could be used. In Brandenburg, a list was made available by the Brandenburg Medical Association, with doctors who had agreed to be contacted. Participants were randomly selected from the address lists. In addition to the questionnaire, the letter was accompanied by an addressed and prepaid envelope. The survey was paper based.

### Statistical analysis

Differences were tested by Chi-Squared test. A *p*-value of 0.05 was interpreted as significantly different. For each question, linear logistic regression analysis was performed to estimate predictors for the answers. Socio-demographic factors and a variable for subjective involvement were used as predictors (see Table 1). Participants were classified as subjectively involved when they responded to “How often do you have contact to patients with multi-resistant organisms in daily work?” with “weekly or more often”. All analyses were performed using SPSS [IBM SPSS statistics, Somer, NY, USA] and SAS [SAS Institute, Cary, NC, USA].

**Table 1:** Demographic characteristics of the participants

Parameter	Responder
Gender, female in percent, n (%)	212 (62.4)
Mean age in years (SD)	51.9 (+/− 8.8)
Mean professional experience in years (SD)	16.7 (+/−10.8)
Medical specialist in percent, n (%)	
General Medicine	288 (84.7)
Internal Medicine	34 (10)
None	5 (1.5)
Other	11 (3.2)
Population of the practice location, n (%)	
< 5,000	56 (16.5)
5,000–19,000	92 (27.1)
20,000–99,000	89 (26.2)
> 100,000	103 (30.3)
Kind of practice, n (%)	
Single practice	193 (56.8)
Joint Practice	113 (33.2)
Practice Communities	26 (7.6)
Patient visits per quartile in percent, n (%)	
< 400	5 (1.5)
400–800	53 (15.6)
801–1,200	128 (37.6)
1,201–1,600	98 (28.8)
> 1,600	48 (14.1)
Contact with patients with MDRO, n (%)	
Weekly or more often	67 (19.7)

Note. All listed parameters were predictors in the multivariable analysis. MDRO multidrug-resistant organism

## Results

The questionnaire was completed by 340 of 987 (34.4%) GPs. The socio-demographic factors are described in Table 1. Most of the participants were female (62.4%). The mean age was 52 (range 33–78) years and the mean work experience was 16.8 years.

### Awareness of antimicrobial resistance

Most of the participants assumed a multifactorial genesis of the rise of multidrug resistant organisms. 80.9% (275/340) of the participants indicated infection control in hospitals, 80.3% (273/340) the use of antibiotics by GPs and 79.1% (261/340) the use of antibiotics in livestock as the main drivers for drug-resistance (multi selection).

The majority of participants (70.2%;239/340) believed that their own prescribing behavior influenced the drug-resistance situation in their area. GPs with longer work experience (> 25 years) assumed less individual influence on drug resistance than do their colleagues with less than 7 years experience as practicing physicians (Odds Ration [OR] 0.32, 95% Confidence Interval [CI] 0.17–0.62; *P* < 0.001).

### Guidelines/source of information

Seven percent (23/340) of the participants stated that there is a lack of good guidelines dealing with antibiotic therapy in ambulatory care. Thirty-nine percent (133/340) of the GPs indicated that they frequently use guidelines for antibiotic therapy. Family doctors under the age of 40 made use of guidelines more often than did those older than 60 (OR 3.97, 95%CI 1.32–11.91; *P* = 0.001). In addition, the location of their place of work (urban vs. rural) influenced the response to this question (Table 2).

**Table 2:** Results of the multivariable analysis

Questions	Odds Ratio (95% CI)
Relevance of antimicrobial resistance for daily work (Answers: strong/medium vs. little/not at all)	
Contacts to patients with MDRO	
Monthly or less frequently	Reference
Weekly or more frequently	5.65 (1.71–18.64)
Do you believe that your prescribing behavior influences the drug resistant organism situation in your area? (Answer: yes vs. no or I don’t know)	
Work experience	
0–7 years	Reference
8–14 years	0.91 (0.44–1-91)
14–25 years	0.44 (0.23–0.85)
> 25 years	0.32 (0.17–0.62)
Do you use guidelines in your daily routine? (Answers: frequently vs. sometimes, seldom or never)	
Population of the practice location	
> 100.000	Reference
20.000–99.000	0.93 (0.50–1.73)
5.000–20.000	1.95 (1.07–3.56)
< 5.000	1.08 (0.53–2.19)
Age (in years)	
> 60	Reference
56–60	3.22 (1.42–7.31)
51–55	1.79 (0.76–4.18)
45–50	2.99 (1.34–6.65)
40–44	3.17 (1.30–7.72)
< 40	3.97(1.32–11.91)
Do you use the strategy of delayed antibiotic prescription? (Answer: very often/often vs. sometimes/seldom/unknown strategy)	
Population of the practice location	
> 100.000	Reference
20.000–99.000	0.49 (0.26–0.91)
5.000–20.000	0.39 (0.21–0.75)
< 5.000	0.57 (0.28–1.17)
Indications for me prescribing antibiotics are … …acute infection with yellow/green sputum (rather yes vs. rather not, to a certain degree)	
Medical specialization	
General Medicine	Reference
Internal Medicine	2.36 (1.08–5.17)
No specialization	9.07 (0.85–96.99)
Work experience	
0–7 years	Reference
8–14 years	1.85 (0.90–3.80)
14–25 years	3.33 (1.69–6.58)
> 25 years	6.54 (3.22–13.30)

*CI* confidence interval, *MDRO* multidrug resistant organism

Note. Predictors in the multivariable analysis are shown in Table 1

### Use of antibiotics

Forty-four percent (151/340) of the GPs stated that one reason for prescribing an antibiotic without a strong indication was that it was just before a weekend when the progression of the infection was difficult to predict. Only 29.4% (74/340) answered that they *often* or *very often* apply delayed prescribing, a strategy for dealing with uncomplicated acute respiratory infections in which the use of an antibiotic is recommended to a patient only if the symptoms persist or worsen or further test results come in. 337 of the 340 participants (99.1%) were familiar with this strategy.

**Fig. 1:**
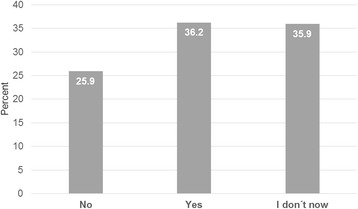
Diagnostic value of the sputum color. Answers on the statement: “For me, the indications for an antibiotic prescription are the green colour of the sputum (in the context of an acute respiratory tract infection)” (*n* = 333)

Thirty-six percent (123/340; Fig. 1) responded that an acute infection with yellow or green sputum is an indication for antibiotic prescription. GPs with more work experience tended to use the color of sputum more often as an indicator for an antimicrobial therapy (> 25 years of working experience vs. < 7 years; OR 6.54, 95% CI 3.22–13.30; *P* < 0.001).

### Awareness of antimicrobial resistance and communication aspects

**Fig. 2:**
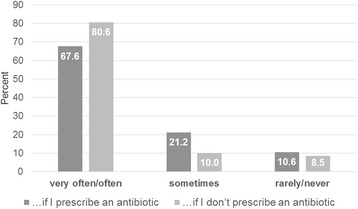
AMR communication. Answers on the Question: “Do you discuss the topic of antimicrobial resistance (AMR) with patients with an acute infection?” (*n* = 338)

**Fig. 3:**
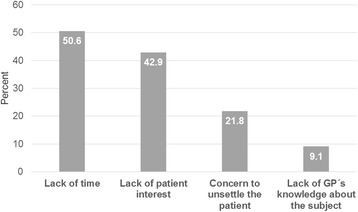
AMR missing communication (multiple selection). *“Reasons not to talk about antibiotic resistance (AMR).”* (n = 338)

Sixty-eight percent (285/340) of the family doctors stated that they often or very often discuss the subject drug-resistant organisms with patients who have an infection that requires antibiotic therapy; 80.6% (274/340) discuss the subject if the patient does not need antibiotics (Fig. 2). In this survey the main reasons for not discussing this topic were a lack of time (50.6%) and the assumption that their patients were not interested in this subject (42.9%; Fig. 3).

## Discussion

We performed a questionnaire-based survey of general practitioners on patterns of antimicrobial use, patient communication, awareness of rising drug-resistance in primary care and the source of information acquisition.

This study demonstrates different specific barriers to rational antibiotic therapy in primary care. First of all, it should be acknowledged that the awareness of antimicrobial resistance among general practitioners has risen during the past few years. In 2007/2008 the Robert Koch Institute distributed a written survey to GPs in Germany. In that survey, 35.8% of the GPs expressed the belief that their prescribing behavior influences drug-resistance in their area [31, 32]. We repeated this question in our survey and 70.2% (239/340) of the participants agreed that their behavior affected drug resistance. This rising awareness might be influenced by international, European and national reports and campaigns that deal with this topic [33–36].

Second, the reliability of sputum color as an indicator for an antimicrobial therapy was overestimated by a majority of the participants. Our investigation found that over one third (36.2%) of the participants use the color as an indication, another third are uncertain (35.9%) and only 25.9% (88/340) of general practitioners do not use sputum color when deciding whether to start antibiotic therapy. Other studies support these findings [28, 37]. In selected diagnoses, especially in chronic lung diseases, the sputum color has a value for antimicrobial therapy decision [38, 39]. Nevertheless, the reproducibility of the evaluation of sputum color has poor inter-rater reliability [40] and is not recommended in the case of an acute respiratory tract infection [41].

In order to work in Germany as a family doctor a 5 year medical specialization in either internal medicine or in general medicine is required. Alternatives to practicing as a general practitioner exist, however rarely. 84.7% (288/340) of the participants have a specialization in general medicine, 10% (34/340) in internal medicine (Table 1). GPs with a specialization in internal medicine were more likely to prescribe an antibiotic based on the color of the sputum than were GPs with a specialization in general medicine (OR 2.36, 95%CI 1.08–5.17; *P* = 0.03). One explanation for why GPs with a specialization in internal medicine were 2.4 times more likely than participants who specialized in general or family medicine to prescribe antibiotics based on sputum color is that the recommendation not to use the color of the sputum as an indicator is very prominent in the guidelines of the German Society of General Practice and Family Medicine (Deutsche Gesellschaft für Allgemeinmedizin und Familienmedizin, DEGAM) [42]. To address the diagnostic uncertainty between a severe acute bronchitis and a starting pneumonia the biomarker as point of care tests are promising [43]. However the reimbursement for GPs in Germany is difficult. An alternative strategy to reduce antibiotic use is delayed prescription [44, 45]. The strategy is well known among the participants (337/340) while only one third (74/340) apply it “often” or “very often”. There is room for improvement in the implementation of the strategy in daily outpatient care, considering the fact that only 21.8% (74/340) use this strategy *often* and only 7.6% (26/340) *very often*.

Third, some authors emphasize that when GPs feel pressure from their patients, they are more likely to prescribe antibiotics [46–49]. Accordingly, about one third of the participants (102/340) prescribe antibiotics when a patient requests some or when the patient wants to return to work quickly (97/340). However, in this survey general practitioners who felt pressure from their patients remained a minority. Unfortunately, we did not conduct patient interviews to evaluate patient requests for antibiotic therapy. Nevertheless, supported by other authors, we believe that GPs place too much importance on patient requests for antimicrobial therapy [50, 51] .

Focusing on aspects of communication, it is striking that a lack of time was the main reason not to talk about antimicrobial resistance (172/340). Some authors describe an antibiotic prescription as an effective means to avoid confrontation and to terminate a consultation [28]. Accordingly, the majority of antibiotic prescriptions are inappropriate in ambulatory care [7]. Patient leaflets could be used for this purpose but there is a lack of established German leaflets for acute respiratory tract infections. There are leaflets of the Center for Disease Control and Prevention (CDC) available in English [52].

This study has certain limitations. All answers are self-reported. Furthermore, as a questionnaire study, this survey contains the risk that respondents will give answers believed to be socially acceptable. The representativeness of the study is limited because the sample was not selected purely randomly, but was instead contacted on the basis of existing address data (self-selection bias). On the other hand, the GPs contacted represent about one third of all GPs working in the region. The response rate is comparable to other studies conducted in Germany [30, 53].

Another limitation is that the questionnaire was only distributed in eastern Germany.

This is due to the fact that this survey was performed in preparation for an intervention campaign that started in August 2016 and focused on rational antibiotic use by GPs in eastern Germany. Looking at the socio-demographic parameters of the participants, it is noticeable that they were about 2 years younger (51.9 vs. 54.3) than the GPs average in Germany and were more likely to be female (62.4% vs 41.0 female GPs). There were only minor differences in the type of workplace [54].

## Conclusion

When deciding on a therapy, the diagnostic value of sputum color is often overestimated. Delayed prescription is well known but only partially applied. Nevertheless, there is a strong awareness of antimicrobial resistance among the participating GPs. Furthermore, time restrictions disturb doctor-patient communication. Implementation of change to a more rational antibiotic use should address such specific barriers as preconditions to having a sustainable effect. This survey shows clear targets for further approaches to reduce the prevalence of drug-resistant organisms.

## Additional file

Additional file 1: English version of the survey questionnaire. (DOCX 28 kb)

## Abbreviations

AMR: Antimicrobial resistance
CI: Confidence interval
GPs: General practitioners
OR: Odds ratio
RAI: Responsible antibiotic use via information and communication
