# Supplementary material for: Antibiotic prescribing behavior among general practitioners – a questionnaire-based study in Germany
Source: BMC Infect Dis. 2018 May 4;18:208. doi: 10.1186/s12879-018-3120-y (PMC5935909; doi:10.1186/s12879-018-3120-y)
Supplement: Supplementary file 1 — English version of the survey questionnaire. (DOCX 28 kb) [file 12879_2018_3120_MOESM1_ESM.docx]

**Table 3.** List of questionnaire items (translated from German)

| **Topic** | **Question** | **Answer categories**^1^ |
| --- | --- | --- |
| **Sociodemographic Data** | **Q1**: Gender | male/female |
|  | **Q2**: Age | In years |
|  | **Q3**: What is the number of inhabitants in your place of work? | >5,000/ 5,000-19,000/ 20,000-99,000/ >100,000 |
|  | **Q4**: Which specialist training did you complete? | general practitioner (GP)/ internal medicine; other |
|  | **Q5**: Type of practice | single practice/ joint practice/ medical care center |
|  | **Q6**: How many visits do you have per quarter? | >400; 400-800/ 801-1,200/ 1,201-1,600/ >1,600 |
|  | **Q7**: Working as a GP since: | in years |
| **Relevance** | **Q8**: How relevant is the subject of antibiotic resistance for your daily work? | highly/ moderately/ sparsely/ not at all |
|  | **Q9**: Do you think, that your antibiotic prescribing behavior influences the antibiotic resistance development within your region? | yes/ no/ don’t know |
|  | **Q10**: How often do you have contact to patients with MDRO during your daily practice? | daily/ weekly/ monthly/ rarer/ never |
|  | **Q11**: What do you think, which sectors should be targeted to slower the development of antibiotic resistances? | *Multiple selection*:   - Hospital hygiene - Animal farm hygiene - Private food hygiene - AB use in hospitals - AB prescriptions by GP - AB intake by patients - AB use in lifestock |
| **Prescribing behavior** | **Q12:** Do you use the strategy of delayed antibiotic prescribing? | Strategy not known/ very often/ often/ sometimes/ rarely/ never |
|  | **Q13**: What are the reasons why antibiotics are prescribed without a hard indication? | *Multiple selection*:   - when the weekend is approaching and the course of the disease is difficult to predict - if the patient wants to get back to work quickly - if the patient demands an antibiotic - if the patient is incompliant - language barriers or cognitive impairments - unknown patient - because further diagnostics are too expensive - to be on the safe side |
|  | **Q14**: Indications for an antibiotic prescription are for me | *Multiple selection*:   - acute infection with white sputum - acute infection with green/yellow sputum - acute exacerbated COPD with little sputum - acute exacerbated COPD with a lot of purulent sputum |
| **Communication** | **Q15a:** Do you discuss the subject of AMR with your patients suffering from infections?  … while prescribing an antibiotic: | very often/ often/ partly/ rarely/ never |
|  | **Q15b:** Do you discuss the subject of AMR with your patients suffering from infections?  … while not prescribing an antibiotic: | very often/ often/ partly/ rarely/ never |
|  | **Q16**: Reasons not to talk about antibiotic resistance (AMR). | *Multiple selection*:   - Lack of time - Concern to unsettle the patient - Lack of patient interest - Lack of GP´s knowledge about the subject |
| **Information sources** | **Q17**: Do you use practice guidelines for antibiotic therapy during your daily work? | frequently/ moderately/ rarely or never/ there are no good guidelines |
|  | **Q18:** Would you like to have more evidence-based therapy guidelines? | yes/ no/ don’t know |
|  | **Q19:** Which are your sources to get current information on antibiotic therapy and ABR? | *Multiple selection:*   - Internet forums - digital information platforms - textbooks - scientific journals - Clinical practice guidelines - direct communication with peer collegues - direct communication with expert - continuing education |
|  | **Q20:** Which additional information sources would be particularly helpful? | *Multiple selection:*   - No further sources needed, existing ones are sufficient - interdisciplinary network - better clinical practice guidelines - better access to existing guidelines - interactive case studies - training games - information and training App - Webpage with news and links - more continuing education without industry sponsoring - e-learning based trainings |

Single selection unless otherwise specified
